# Metal-Support Cooperative Effects in Au/VPO for the Aerobic Oxidation of Benzyl Alcohol to Benzyl Benzoate

**DOI:** 10.3390/nano9020299

**Published:** 2019-02-20

**Authors:** Sebastiano Campisi, Michele Ferri, Carine E. Chan-Thaw, Felipe J. Sanchez Trujillo, Davide Motta, Tommaso Tabanelli, Nikolaos Dimitratos, Alberto Villa

**Affiliations:** 1 Università degli Studi di Milano, Dipartimento di Chimica, Via C. Golgi 19, 20133 Milano, Italy; sebastiano.campisi@unimi.it (S.C.); michele.ferri@unimi.it (M.F.); 2 Institut pour la Maîtrise de l’Énergie–Université d’Antananarivo BP 566, 101 Antananarivo, Madagascar; carine.chanthaw@gmail.com; 3 Cardiff Catalysis Institute, School of Chemistry, Cardiff University, Main Building, Park Place, Cardiff, CF10 3AT, UK; SanchezF@cardiff.ac.uk (F.J.S.T.); MottaD@cardiff.ac.uk (D.M.); 4 Dipartimento di Chimica Industriale e dei Materiali, ALMA MATER STUDIORUM Università di Bologna, Viale Risorgimento 4, 40136 Bologna, Italy; tommaso.tabanelli@unibo.it

**Keywords:** gold, vanadyl pyrophosphate oxide (VPO), alcohol oxidation, esterification

## Abstract

This paper studies the cooperative effect of Au nanoparticles deposited on vanadyl pyrophosphate oxide (VPO) in the liquid phase oxidation of benzyl alcohol. VPO was prepared using the classical method by thermally treating VOHPO_4_·0.5H_2_O precursor in reacting atmosphere at 420 °C for a period of 72 h. Au nanoparticles were deposited by incipient wetness method. The catalysts were characterized by means of XRD, TEM, XPS and Raman. The bulk VPO catalyst contains vanadyl pyrophosphate phase ((VO)_2_P_2_O_7_), and a small amount of VOPO_4_. The catalytic system exhibits a high activity in the base-free liquid phase oxidation of alcohols compared to Au on activated carbon, classic catalyst used for this type of reaction. Au/VPO showed a high peculiar selectivity to benzyl benzoate (76%), an important product used in the pharmaceutical and perfume industries. This behavior might be ascribed to the presence of strong acid sites of VPO, as determined by liquid phase titration. Stability tests performed on Au/VPO showed a deactivation of 10% after the first run, but a constant conversion along the following five cycles. This phenomenon can be attributed to the increase of mean Au particle size (from 19.1 to 23.4 nm) after recycling tests as well as the partial leaching of Au and V in the reaction media. Moreover, XRD evidenced a modification in the VPO structure with the partial formation of VOHPO_4_·0.5H_2_O phase.

## 1. Introduction

The liquid phase oxidation of benzyl alcohol, using heterogeneous catalysts and oxygen as oxidant, has been extensively studied in the last years [1,2,3]. Depending on the catalytic materials and the reaction conditions (temperature (60–160°C), solvent, oxygen pressure), many products of industrial interest, such as benzaldehyde, benzyl benzoate, benzoic acid, and benzyl ether can be obtained [2]. For example, benzaldehyde is used as flavoring agent and as precursor for the production of pharmaceuticals or plastic additives [4,5]. Benzyl benzoate finds applications as topical treatment for human scabies [6], as well as dye carrier [7]. Different mechanisms for benzyl benzoate formation have been proposed, including the subsequent reaction of benzaldehyde via acid catalyzed reactions to give benzyl benzoate and its dibenzyl acetal or the esterification of benzoic acid by the substrate [8,9,10,11,12]. 

Au catalysts are widely investigated as catalyst in the liquid phase oxidation because of their resistance against deactivation [13,14,15,16,17]. The drawback related to the use Au catalyst is the need to operate under alkaline conditions, which facilitate the first step of the oxidative dehydrogenation (H abstraction), however making purification a challenge and limiting the industrial application [18,19]. Recent studies have shown that the support can play a key role in enhancing the activity and the selectivity of the gold catalyzed reactions, even in absence of a base [20,21,22,23,24]. Indeed, the support surface properties can tune the interaction with the metallic particles, modifying both the catalysts electronic and structural properties. In addition, the supports can also be active in the specific reaction increasing the synergistic effect with the active sites of the metal nanoparticles [25].

Vanadium phosphate oxide (VPO) based heterogeneous catalysts have been reported to be active in liquid phase reactions [26]. The selective oxidation of *n*-butane, instead of benzene, to maleic anhydride is catalyzed by VPO catalysts composed mainly of vanadyl pyrophosphate ((VO)_2_P_2_O_7_ with high conversion and good selectivity. VPO was reported to be effective in biomass conversion [27], hydrocarbon oxidations [28], and benzyl alcohol oxidation [29,30]. Therefore, we decided to study the properties of VPO as support on the catalytic behavior of Au nanoparticles in the benzyl alcohol oxidation. We recently showed that the combination of Au and VPO results in a synergistic effect when used for the gas phase *n*-butane oxidation to maleic anhydride [31]. In the present study, we demonstrate that the activity of Au/VPO system is significantly higher than the one of Au/AC, the classical Au based catalyst used for the liquid phase reactions. Moreover, a peculiar selectivity to benzyl benzoate was observed. To correlate structure and activity, the catalysts were characterized by means of characterization techniques, such as XRD, TEM, XPS, and Raman.

## 2. Materials and Methods

Catalysts preparation: VPO catalysts were prepared according to the following procedure. V_2_O_5_ (10 g, Aldrich (St. Louis, MO, USA)) and H_3_PO_4_ (85 wt%, 12.9 g, Aldrich (St. Louis, MO, USA)), dissolved in an aqueous solution were refluxed for 72 h to form a yellow slurry containing the VOPO_4_·2H_2_O precursor. The water was evaporated and the cake dried for 12 h at 120 °C. Afterwards, the formation of VOHPO_4_·0.5H_2_O (precursor) was accomplished by suspending VOPO_4_·2H_2_O in isobutanol and refluxing for 72 h. The whitish-blue slurry was filtered and the resulting paste was dried at 120 °C overnight. The P/V atomic ratio was 1.0. The precursor was then thermally treated under a stream of propylene, oxygen and nitrogen (propylene/O_2_/N_2_ = 5.2/18.9/75.9 vol.%, 29 mL/min^−1^) at 420 °C for a period of 72 h (after this period samples are referred as fresh catalysts), and finally resulted in the formation of the desired VPO phase. Activated carbon (AC) was obtained from Camel (X40S; Surface area A = 1100 m^2^/g; Pore volume = 1.5 mL/g; generated pH 7.5). The 1 wt% Au_IW_/VPO and 1% Au_IW_/AC samples were prepared by incipient wetness method. The desired amount of HAuCl_4_ (Au = 0.051 mmol) was dissolved in water (volume = pore volume of the chosen support) impregnated onto the support (1g). The paste was ground and dried at 150 °C overnight and, finally, thermally treated as described above. 1% Au_SI_/AC was prepared by sol—immobilization method following the procedure reported by Wang et al. [32].

Catalytic tests: alcohol oxidation was performed in a 30 mL glass reactor equipped with a thermostat and an electronically controlled magnetic stirrer. The glass reactor was connected by tubing to a mass-flow controller used to flow gas mixtures. Benzyl alcohol and the catalyst were using *p*-xylene as solvent. The benzyl alcohol/metal ratio was typically approximately 1000 moles alcohol per mole Au. Alcohol/ xylene ratio of 25/75 by percent volume was used during experiments with the total liquid volume always kept at 10 mL. The reactor temperature was set to 120 °C and 2 bar of O_2_. The desired temperature was reached in few minutes, and reaction time zero was marked by the beginning of stirring. Periodic removal of samples from the reactor was performed by a syringe. Identification and analysis of the products were done by comparison with reference samples by gas chromatography using a HP 7820A gas chromatograph equipped with a capillary column (HP-5 30 m × 0.32 mm, 0.25 mm film, made by Agilent Technologies, Santa Clara, CA, USA) and a thermal conductivity detector. Quantification of reaction products was done by internal calibrations on the gas chromatograph using undecane as a standard. Hot filtration test was performed by filtrating the catalyst after 6h without cooling the reaction mixture. The filtered solution was immediately charged in the reactor and maintained under the reaction conditions for additional 6h and samples were analyzed after an additional hour to check any changes in the catalytic activity and product distribution.

Characterization of catalysts: the characterization of the catalysts was performed using XRD, XPS, Raman, acid site titration in liquid phase and TEM techniques. X-ray diffraction (XRD) patterns were collected using a Rigaku D III-MAX horizontal scan powder equipped with a graphite monochromator, operating at 40 kV and 40 mA, and employing nickel-filtered CuKα radiation (λ = 0.1542 nm). The calculation of the crystallite size of the (2 0 0) plane is given by the Scherrer equation: t = 0.9λ/(β_h k l_ × cos θ_h k l_), where t is the crystallite size, λ is the X-ray wavelength of radiation for CuKα, β_h k l_ is the full-width at half maximum (FWHM) at (h k l) peak and θ_h k l_ is the diffraction angle. X-ray photoelectron spectroscopy (XPS) was performed on a Thermo Scientific K-alpha+ spectrometer. Samples were analyzed using a monochromatic Al x-ray source operating at 72 W (6 mA × 12 kV), with the signal averaged over an oval-shaped area of approximately 600 × 400 microns. Data were recorded at pass energies of 150 eV for survey scans and 40 eV for high resolution scan with a 1eV and 0.1 eV step size respectively. Charge neutralization of the sample was achieved using a combination of both low energy electrons and argon ions (less than 1 eV) which gave a C(1s) binding energy of 284.8 eV.

All data were analyzed using CasaXPS (v2.3.17 PR1.1) using Scofield sensitivity factors and an energy exponent of −0.6. Raman spectroscopy was performed with a Renishaw inVia Raman microscope. Bare supports, fresh and used catalysts were analyzed. Typically, a sample of approximately 0.01 g was placed on a metal slide inside the spectrometer. The powder was analyzed under an IR class laser (514 nm) with a laser intensity of 50%. The sample was scanned at an attenuation time of 22 s and 10 scans were carried out to give a spectrum. 

Surface acid sites of VPO and Au/VPO sample were measured in liquid phase by titration with solutions of 2-phenylethylamine (PEA). Titrations were carried out in cyclohexane.

VPO-based samples (ca. 0.05 g, crushed and sieved as 25–45 mesh particles) was placed in a sample holder (stainless steel tube, 2 mm diameter and 12 cm of length) between two sand pillows. Samples underwent then thermal pre-treatment (150 °C in 8 mL min^−1^ air flux for 4 h) and successively the tube was filled with the solvent (cyclohexane). The sample holder was then mounted on a recirculation chromatographic line (HPLC), equipped with a Waters 515 pump and a monochromatic UV detector (Waters, model 2487, Sesto San Giovanni, Italy, working at fixed = 254 nm). During the analyses, the sample was maintained at constant temperature (30.0 ± 0.1°C). By means of successive injections of dosed amounts of PEA (50 μL, ca. 0.10 M in cyclohexane) into the line, a step-chromatogram was obtained, where each step represents the achieving of adsorption equilibrium. After the collection of the first adsorption isotherm on fresh sample (I° run), pure solvent was flowed through the saturated sample overnight (ca. 16 h), thus permitting desorption of the probe molecules from weakly interacting sites; then, a new adsorption of PEA on the same sample was repeated (II° run) to quantify strong acid sites. The numerical interpretation of the collected data in Supporting Information has been performed as reported elsewhere [33]. Previous tests of PEA adsorptions on sea sand showed that the probe molecule were adsorbed in negligible amount.

Acid–base titration measurements of AC was performed using a Mettler Toledo titrator equipped with a DGi 114-SC electrode. Typically, the sample (100 mg) was dispersed in the KCl solution (50 mL, 10^−3^ m). The mixture was kept under vigorous stirring overnight. Prior to measurement, the mixture was degassed bubbling N_2_ for at least 1 h until the pH value was constant.

Particle size distributions and mean particle size were obtained by means of transmission electron microscopy (TEM) using a JEOL JEM 2100 TEM, Akishima, Tokyo, Japan operating at 200 kV. Samples for examination were prepared by dispersing the catalyst in high purity ethanol. A drop of the suspension was allowed to evaporate on a holey carbon film supported by a 300-mesh copper TEM grid. Samples were subjected to bright field diffraction contrast imaging experiments. Mean particle sizes and particle size distributions were determined by measuring the size of over 200 particles from different selected areas. The metal content was checked by Atomic Absorption Spectroscopy (AAS) analysis of the filtrate, on a Perkin Elmer 3100 instrument, Waltham, Massachusetts, USA.

Calculations of the number of exposed surface atoms were performed by assuming that all the nanoparticles had cub-octahedral morphology with cubic close-packed structure in this size range, the model of full-shell nanoparticles was adopted [34]. The total number of the Au atoms in the cluster for a given cluster size can be calculated using the following equation (1):

d_sph_ = 1.105 d_at_N_T_^1/3^ (1)

where d_sph_ is the mean diameter of the Au particles obtained from TEM analysis and d_at_ is the atom diameter of Au, 0.288 nm. The number of surface atoms (N_s_) and n can be calculated from equations (2) and (3), based on the values of N_T_:

N_T_ = (10n^3^ − 15n^2^ + 11n – 3)/3 (2)

N_s_ = 10n^2^ − 20n + 12 (3)

Calculation of Activity based on the surface atoms

The TOF based on the surface atoms can then be calculated as follows:

% of fraction of atoms lying at the surface:

A = (N_s_/N_T_) × 100 (4)

TOF based on N_s_ = TOF(calculated for bulk gold using the nominal weight)/A [35].

## 3. Results

VPO was prepared according to the procedure reported by Luciani et al. [31]. 1% Au was added by incipient wetness (IW) to VPO and for comparison to activated carbon, typical support used for liquid phase reactions. The 1% loading was confirmed by Atomic Absorption Spectroscopy (AAS). Au nanoparticles size was calculated by Transmission Electron Microscopy (TEM) analysis, showing a mean diameter of 19.1 nm and 23.1 nm for Au_IW_/VPO and Au_IW_/AC (Table 1), respectively. The deposition of Au on VPO resulted in a slight decrease in the acidity of the support both in terms of number of acid sites and relative strength (Table 1). Actually, when liquid-phase titration was performed in cyclohexane (aprotic solvent with negligible polarity) using phenylethylamine (PEA) as a basic probe (Figure S1), VPO confirmed its well-known acidic character, possessing 0.485 mmol g^−1^ acid sites, which almost all were strong sites. After the Au introduction, the total number of acid sites decreased to 0.324 mmol g^−1^ and also the relative amount of strong acid sites was slightly lower (92%). This kind of acid site determination in a liquid environment allowed to probe the surface acidity under conditions close to the experimental ones used in the catalytic tests (*effective acidity*). Previous works confirmed the accuracy of the method and the agreement with other conventional techniques (NH_3_-TPD, Infrared Spectroscopy) usually employed for acidity measurements [36,37].

The catalysts were evaluated in the benzyl alcohol oxidation using O_2_ as oxidizing agent (benzyl alcohol/xylene 25/75 per cent volume, alcohol/Au = 1000 mol/mol, 2 atm O_2_, T= 120 °C) and both activity and selectivity are reported for each system in Table 1. Xylene has been chosen as solvent due to its low vapor pressure and low reactivity [8]. Reaction conditions have been optimized to confirm to operate under kinetic regime (Tables S1–S2).The activity was calculated firstly based on the moles of alcohol converted per hour per total mol of metal. Au_IW_/AC and Au_SI_/AC were used as model catalysts to compare catalytic activity and selectivity with the Au/VPO analogues.

**Figure 1:**
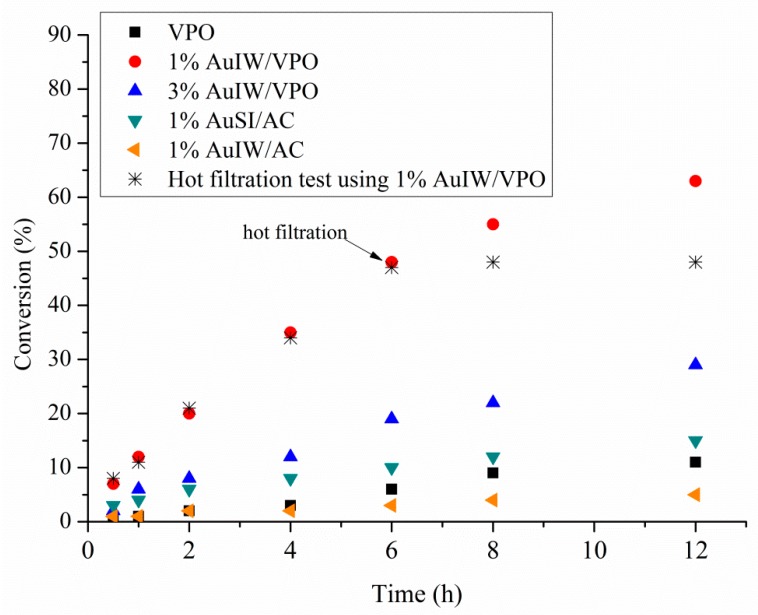
Reaction profile of un-promoted and promoted VPO catalysts with Au in the benzyl alcohol oxidation.

According to literature reports, VPO is an active catalyst for the liquid phase oxidation of alcohols [29,30]. Also in our study, VPO is active (Table 1) reaching a conversion of 9% after 12 h (Figure 1). The addition of Au to VPO increased significantly the catalytic activity by a factor of 7 in terms of conversion, with an initial activity of 120 (Mol of alcohol converted per hour per mol of Au) (Table 1) and a conversion of 64% after 12 h (Figure 1). On the contrary, Au deposited by incipient wetness (IW) on activated carbon showed a low activity (8 Mol of alcohol converted per hour per mol of Au), comparable to the one of bare VPO (Table 1). Au_IW_/VPO and Au_IW_/AC catalysts were compared to Au/AC catalyst prepared by sol immobilization (Au_SI_/AC), a system largely used to prepare small Au NPs (3.6 nm) even on carbon (Table 1). Au_SI_/AC showed a higher activity than Au_IW_/AC (27 and 8 Mol of alcohol converted per hour per mol of Au, respectively), following the trend that smaller Au NPS are more active than larger ones [38]. However, the activity of Au_SI_/AC was still remarkably lower than the one of Au_IW_/VPO (27 and 120 Mol of alcohol converted per hour per mol of Au, respectively) (Table 1). To exclude Au particle size effect, the activity was also calculated based on the total number of surface Au atoms (Ns) (Table 1). The results confirmed the synergistic effect of Au and VPO despite the large Au particle size. 

In terms of selectivity, (Table 1), it was found that the support (VPO) has a strong influence on the selectivity of Au/VPO system. A selectivity of 83% and 82% to benzaldehyde was obtained for Au_IW_/AC and Au_SI_/AC (mean particle size of 19.0 and 3.6 nm, respectively Au in the presence of metallic state), with benzoic acid and benzyl benzoate as minor by-products (Table 1). On the contrary, Au_IW_/VPO, promoted the formation of benzyl benzoate (76%) as major product, together with benzaldehyde (8%) and benzyl ether (6%) (Table 1). The peculiar selectivity seems to be related to the presence of VPO as support. Indeed, pure VPO showed also a high selectivity to benzyl benzoate (78%).

**Figure 2:**
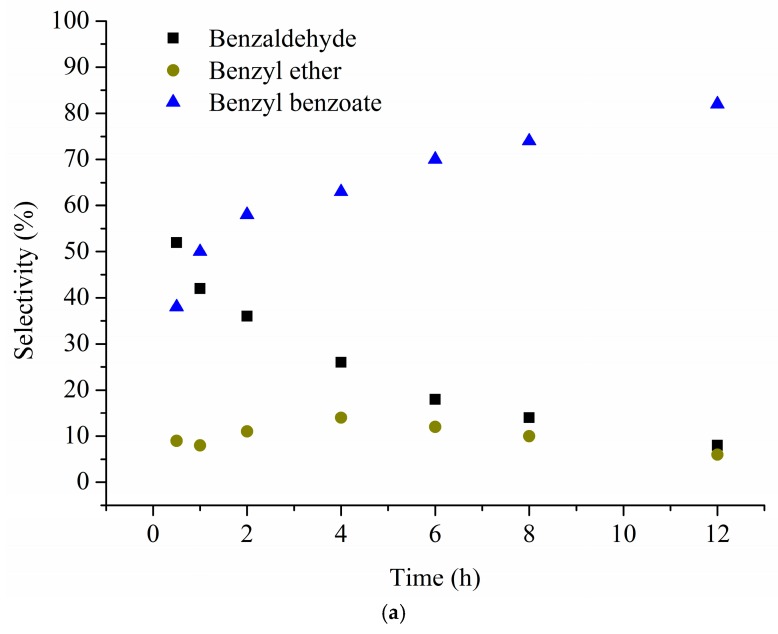

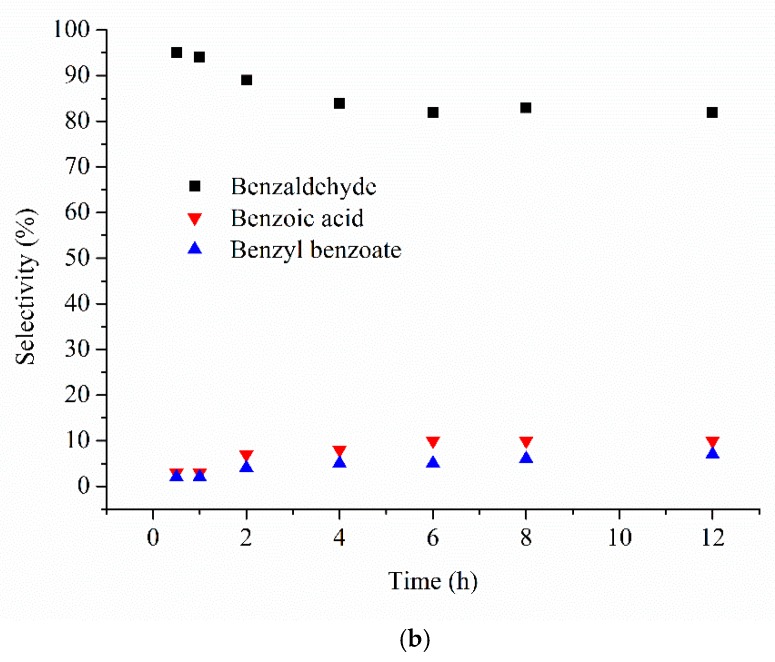
Products distribution for (**a**) 1% Au_IW_/VPO and (**b**) 1% Au_IW_/AC catalysts.

**Scheme 1:**
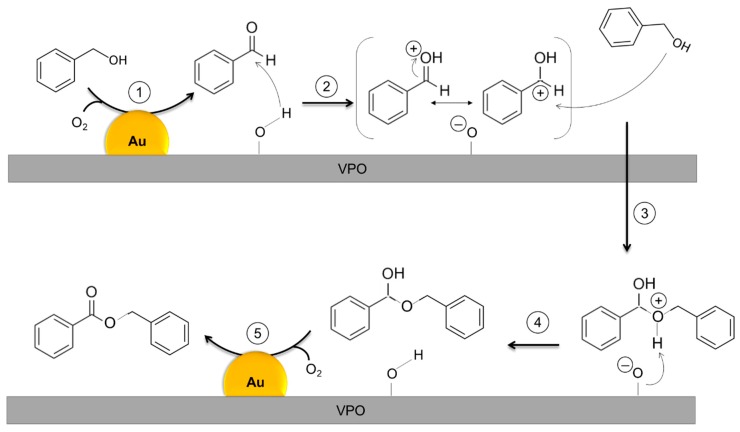
Proposed reaction pathway for benzyl benzoate formation from benzyl alcohol.

Observing the reaction profile versus time one stream (Figure 2a), the increase in the selectivity to benzyl benzoate, using Au_IW_/VPO as catalyst, is associated to a decrease of benzaldehyde. The selectivity to benzyl ether remained constant. In this case, benzyl benzoate seems to be formed by the subsequent reaction of benzaldehyde to give benzyl benzoate and its dibenzyl acetal as proposed by Li et al. [12]. This reaction pathway (Scheme 1) could be promoted by the strong acidity of the VPO support.

**Figure 3:**
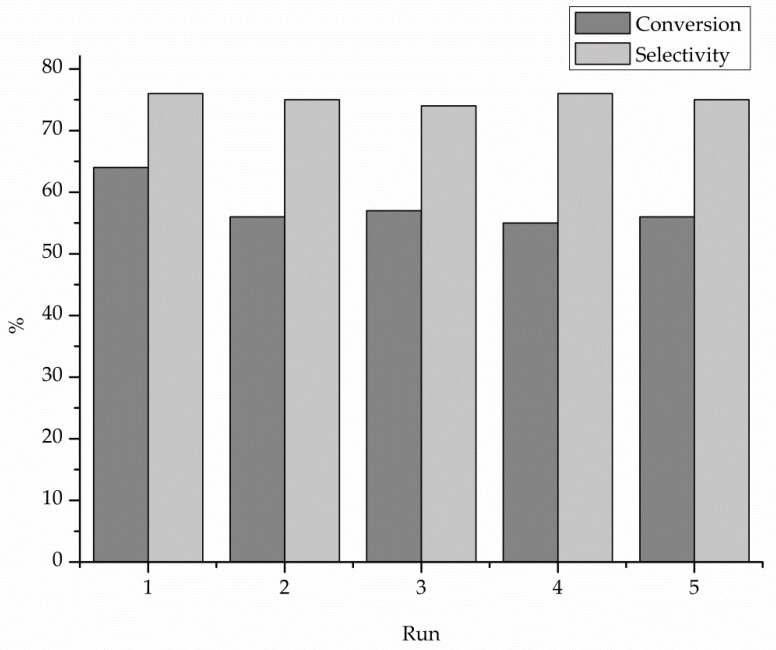
Recycling tests using 1% Au_IW_/VPO catalyst.

Au_IW_/AC, which does not contain strong acid sites (generated pH of AC = 7.5) showed a different reaction profile (Figure 2b). The selectivity to benzaldehyde, benzoic acid and benzyl benzoate showed a minimal variation during the reaction. The stability of Au_IW_/VPO catalyst was also evaluated in a recycling experiments (time of reaction 12 h), in which the catalyst of the first run was recovered by centrifugation and reused in the following run without any further purification, (Figure 3). The catalyst resulted to be stable both in terms of activity and selectivity. Indeed, besides a slight 10% loss of activity after the first run, the catalyst maintained a constant conversion along the five runs, without any significant variation in the selectivity.

TEM, XPS, X-ray diffraction (XRD), AAS and Raman analysis were carried out for the as-prepared and used catalysts to provide insight into the loss of activity observed with recycling after the first run. 

**Figure 4:**
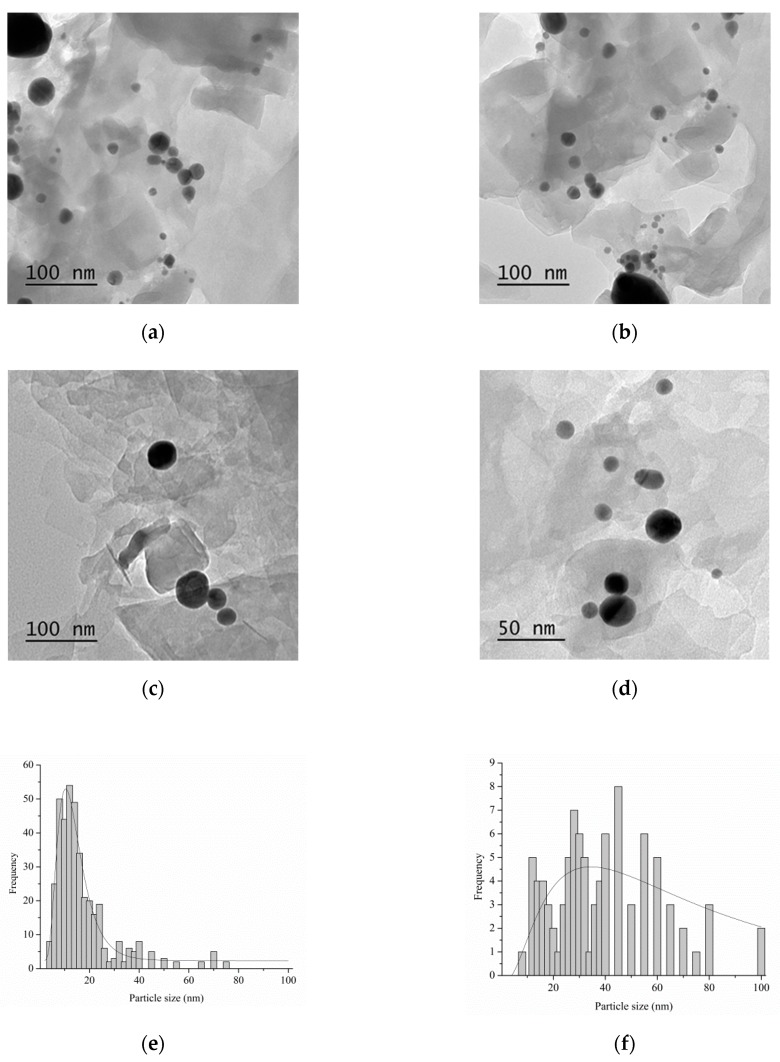
TEM overview of (**a,b**) fresh and (**c,d**) used (after five cycles) and particles distribution of (**e**) fresh and (**f**) used 1% Au_IW_/VPO catalysts.

TEM analysis demonstrated that after the reusability tests (five cycles), the Au nanoparticles on VPO had coarsened from 19.1 to 23.4 nm, which indicates agglomeration/sintering under reaction conditions (Figure 4).

**Figure 5:**
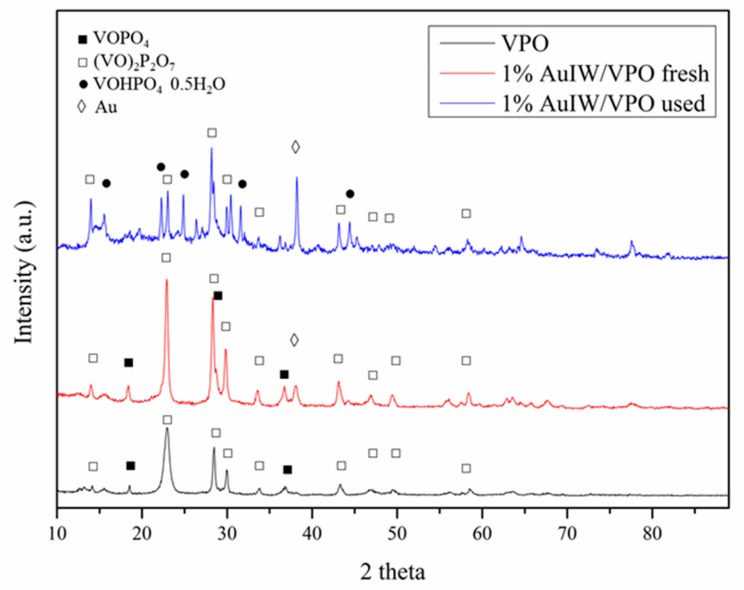
XRD patterns of fresh and used (after five cycles)1% Au_IW_/VPO catalysts.

X-ray diffraction data (XRD) evidenced differences in the structure of fresh and used (after five cycles) Au_IW_/VPO catalysts. The XRD pattern of the fresh VPO and Au_IW_/VPO indicated the presence of characteristic peaks of (VO)_2_P_2_O_7_ phase (JCPDS-01-089-8338) (Figure 5), at 2θ = 14.4°, 23.2°, 28.8°, 30.1°, 37.2°, 43.9°, 46.7°, 49.7°,58.7°, with three main characteristic peaks at 2θ = 23.2°, 28.8°, 30.1°, which correspond to (2 0 0), (0 2 4) and (0 3 2) planes, respectively [39]. The crystallite sizes of the (2 0 0) was calculate to be 18 and 21 nm for VPO and AuIW/VPO, respectively. for Peaks at 2θ = 18.9°, 29.0° and 34.2° evidenced the presence of VOPO_4_ ·2H_2_O phase [39]. In the case of Au_IW_/VPO the typical diffraction peak for gold in the metallic state (2θ = 38.3°) corresponding to (1 1 1) plane was observed (JCPDS-00-002-1095). Additional peaks were observed on the XRD pattern of the used Au_IW_/VPO catalyst (Figure 2). These new diffraction peaks 2θ = 22.4°, 26.4° and 31.6° are characteristic of the presence of VOHPO_4_·0.5H_2_O [40]. The presence of the corresponding diffraction peaks implies a partial modification of the structure of the catalyst during the reaction. These modifications are probably due to the formation of water during the benzyl alcohol dehydrogenation [8]. The diffraction peaks corresponding to VOPO_4_·2H_2_O were less evident on the used catalyst, (Figure 5).

**Figure 6:**
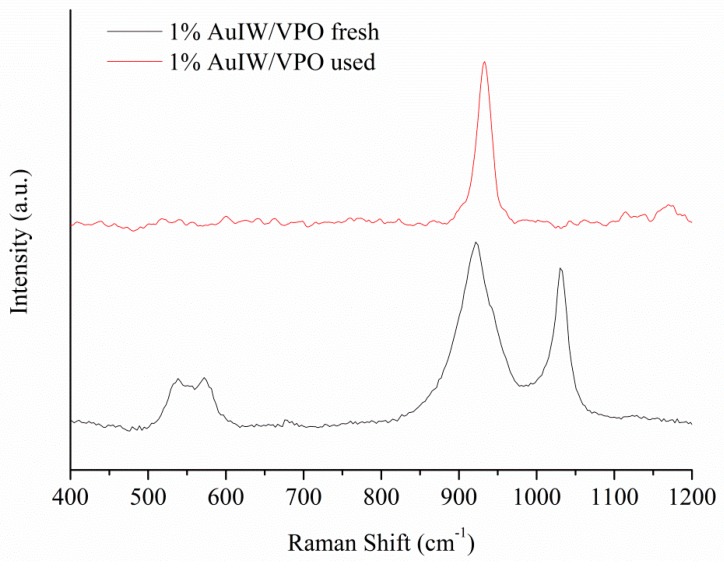
Raman spectra of fresh and used 1% Au_IW_/VPO catalysts.

Figure 6 shows the Raman spectra of the fresh and used Au_IW_/VPO catalysts. For the fresh samples, the band at 945 cm^−1^ could be attributed to the presence of (VO)_2_P_2_O_7_ and the additional band at 542, 573 and 1058 cm^−1^ to the presence of VOPO_4_. After reaction the bands attributable to VOPO_4_ disappeared. These results are in good agreement with XRD measurements.

**Figure 7:**
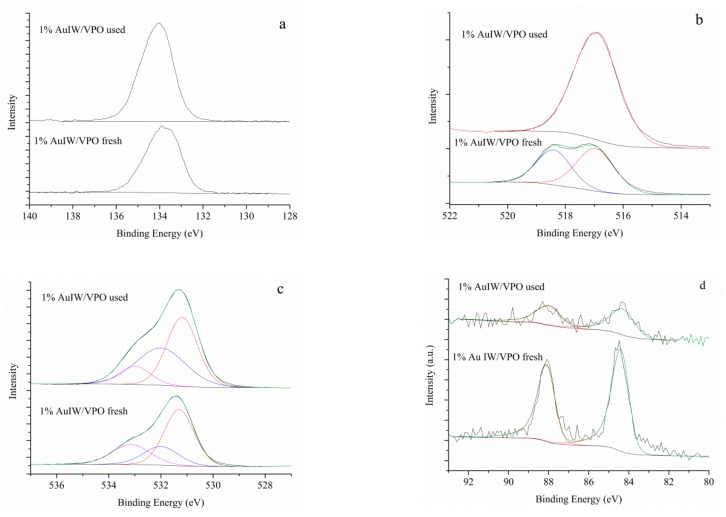
XPS Spectra of the (**a**) P1s, (**b**) V2p, (**c**) O1s and (**d**) Au4f regions of fresh and used 1% Au_IW_/VPO catalysts.

X-ray photoelectron spectroscopy (XPS) analysis of the different fresh and used Au_IW_/VPO catalysts was performed to investigate the surface chemistry of the catalysts. The chemical species present on the surface and their relative amount are summarized in Table 2 and Figure 7. V 2p peak of the fresh catalyst showed two main peaks at BE of 517.0 and 518.4 eV corresponding to V^4+^ and V^5+^ [41,42,43], respectively. After reaction, only the peak corresponding to V^4+^ (BE 516.9 eV) was present, confirming the disappearance of VOPO_4_ structure. The binding energy of P 2p were 134.1 and 133.8 eV, for fresh and used catalysts respectively. O 1s peak revealed the presence of three peaks with binding energies of 531.3 eV, 532.0 eV and 533.2 eV, respectively. The first two peaks correspond to lattice oxygen ions in vanadium phosphates, whereas the third one is related to the presence of surface hydroxide ions and carbonates [44,45]. BE and relative amount of oxygen species remained similar after catalytic tests.

The gold XPS spectra (Au4f region) collected for the catalysts showed Au4_f7/2_ BE at 84.3 and 84.2 eV for fresh and used Au_IW_/VPO, respectively (Table 2, Figure 7). Au for the fresh sample is therefore slightly in a positive oxidation state (Au^δ+^), whereas the used sample is mainly present in the metallic state. However, the lower relative amount of Au at the surface present after reaction (0.14 and 0.03 before and after reaction, respectively) suggests that (i) part of Au was leached in the reaction solution, (ii) Au is partially covered by products deriving from benzyl alcohol oxidation and (iii) increase of Au mean particle size.

Finally, AAS analysis performed on the reaction solution after the recycling tests showed a partial leaching of Au (2%) and V (3%). Considering the aforementioned data, we carried out a hot filtration experiment to verify the role of the leached species (V and Au) into the reaction media. As it is shown in Figure 1, the hot filtration experiment showed that after the filtration negligible increase of catalytic activity was observed, confirming the heterogeneous active role of the Au/VPO catalyst and the leached species did not contribute to the observed activity.

## 4. Discussion

The observed catalytic behavior for Au/VPO can be related to a synergistic effect between Au and VPO. Au catalyst shows typically low activity in the base free alcohol oxidation. Indeed, Au requires a basic environment for the first step of the oxidative dehydrogenation (H-abstraction). However, the support can also play an important role in the initial activation of the alcohol that might occur on the catalyst surface, enhancing the activity of Au systems. Studies performed on VPO for *n*-butane oxidation, revealed that the high reactivity of VPO results from the combination of the Lewis acid sites (i.e., V^4+^) able to perform hydride abstraction, and the Brønsted acid sites which favor the stabilization of the reaction intermediates and adsorbed oxygen species [46]. In fact, liquid-phase titration of surface acid sites evidenced the high number of strong acid sites present at the surface of Au/VPO. Furthermore, the peculiarity of Au/VPO lies in its ability to produce benzyl benzoate, whereas Au/AC systems show benzaldehyde as the major product. The presence of strong acid sites on VPO is probably responsible of the ester formation. Indeed, Barbosa et al., demonstrated that a combination of strong Lewis and Brønsted acids present on silica modified with H_2_SO_4_, facilitated the formation of benzyl benzoate via acid protonation [47]. Moreover, based on the XPS analysis the surface P/V ratio for pure VPO, fresh and used Au/VPO samples were 3.3, 3.4 and 2.9, respectively (Table 2). These values indicate surface P enrichment and it is considered that the excess of surface phosphorous with respect to the bulk atomic ratio of 1 for P/V will facilitate the increase of the surface content of POH groups. The presence of the corresponding surface Brønsted POH groups are a clear evidence of the existence of Brønsted acid sites, in agreement with quantitative data from liquid-phase titration [46]. Moreover, it has been shown previously that the increase of number of acid sites is related to the enrichment of the surface by P and the amount of ammonia adsorbed by performing TPD-NH_3_ studies_,_ as a function of P/V surface ratio can follow a linear increase [41]. These results indicate that the presence of strong acid sites can have a significant impact for enhancing selectivity to benzyl benzoate via hemiacetal species [12,47]. In the near future we will focus to understand better and discriminate the specific role of Brønsted and Lewis acid sites in the un-promoted and promoted VPO catalysts.

## 5. Conclusions

Au nanoparticles were deposited by incipient wetness method on VPO prepared by thermally treating VOHPO_4_·0.5H_2_O precursor in propylene/oxygen/nitrogen at 420 °C for a period of 72 h. XRD, XPS and Raman showed that VPO catalyst contains vanadyl pyrophosphate phase ((VO)_2_P_2_O_7_), and a small amount of VOPO_4_. The surface P enrichment revealed by XPS resulted in the insurgence of strong acid sites, as determined by liquid-phase titration. The catalyst exhibited high activity in the base-free liquid phase oxidation of benzyl alcohols compared to Au on activated carbon. Au/VPO showed a high peculiar selectivity to benzyl benzoate (76%) due to the presence of acid sites on the support. Recycling tests, performed to study the stability of Au/VPO system, showed a deactivation of 10% after the first run, but a constant conversion along the following five cycles. This phenomenon was attributed to the growth of Au particles (increase of Au mean particle size from 19.1 to 23.4 nm), after recycling tests as well as the partial leaching of Au and V in the reaction media. Moreover, XRD patterns highlighted a modification in the VPO structure with the minor formation of VOHPO_4_·0.5H_2_O phase.

## Figures and Tables

**Table 1:** Activity and selectivity of Au-based catalysts in the benzyl alcohol oxidation.

Catalyst ^1^	Au Size (nm)	Acid sites ^2^	Activity ^3^	Activity Ns ^4^	Selectivity (%) ^5^			
		(mmol g^−1^)			Benzal-Dehyde	Benzyl Ether	Benzyl Benzoate	Benzoic Acid
VPO ^6^	-	0.485 (>99%)	2	-	16	4	78	-
1%Au_IW_/VPO	19.1	0.324 (92%)	120	1639	8	6	76	-
1%Au_SI_/AC	3.6	-	27	98	82	1	7	7
1%Au_IW_/AC	23.1	-	8	114	83	-	6	9

^1^ Reaction conditions. Alcohol/Au: 1000, T = 120 °C, pO_2_ = 2 atm, benzyl alcohol/xylene 25/75 per cent volume. ^2^ Surface acid sites measured with PEA probe in cyclohexane at 30 °C. Strong acid sites in percent are indicated in parenthesis. ^3^ Mol of alcohol converted per hour per total number of moles of Au calculated after 0.5 h of reaction. ^4^ Mol of alcohol converted per hour per total number of surface Au atoms (Ns) after 0.5 h of reaction. ^5^ Selectivity after 12 h of reaction. ^6^ For VPO test, the same amount of catalyst as for the 1% Au/VPO was used.

**Table 2:** XPS of 1% Au_IW_/VPO fresh and used catalysts.

Samples		V2P		P1s	O1s			Au4f	Au %at	P/V
		V^4+^	V^5+^							
VPO	BE eV	517.0	518.2	134.0	531.3	532.1	533.3	-	-	3.3
	%	*58.2*	*41.8*	*100*	*54.7*	*29.6*	*15.7*			
Au_IW_/VPO fresh	BE eV	517.0	518.4	134.1	531.3	532.0	533.2	84.3	0.14	3.4
	%	*57.6*	*42.4*	*100*	*55.1*	*30.6*	*14.3*	*100*		
Au_IW_/VPO used	BE eV	516.9	-	133.8	531.2	532.0	533.0	84.2	0.03	2.9
	%	*100*	*-*	*100*	*60.0*	*21.3*	*18.7*	*100*		

## Supplementary Materials

The following are available online at http://www.mdpi.com/2079-4991/9/2/299/s1, Figure S1: PEA adsorption isotherms, collected in cyclohexane at 30 °C, for VPO (a) and Au-VPO (b). Experimental data were fitted using Langmuir model equation. Strong acid sites Langmuir isotherm was obtained by mathematical difference between first and second run of adsorption, Table S1: Optimization of reaction parameters using 1% AuIW/VPO: Alcohol/Au ratio, Table S2: Optimization of reaction parameters using 1% AuIW/VPO: pO_2_.
