# Supplementary material for: Metal-Support Cooperative Effects in Au/VPO for the Aerobic Oxidation of Benzyl Alcohol to Benzyl Benzoate
Source: Nanomaterials (Basel). 2019 Feb 20;9(2):299. doi: 10.3390/nano9020299 (PMC6410264; doi:10.3390/nano9020299)
Supplement: Supplementary file 1 [file nanomaterials-09-00299-s001.pdf]

Supporting Information

Metal-Support Cooperative Effects in Au/VPO for the Aerobic Oxidation of Benzyl Alcohol to Benzyl Benzoate

Sebastiano Campisi [1], Michele Ferri [1], Carine E. Chan-Thaw [2], Felipe J. Sanchez Trujillo [3], Davide Motta [3], Tommaso Tabanelli [4], Nikolaos Dimitratos [4,*] and Alberto Villa [1,*]

1 Università degli Studi di Milano, Dipartimento di Chimica, Via C. Golgi 19, 20133 Milano, Italy; Sebastiano.Campisi@unimi.it (S.C.); Michele.Ferri@unimi.it (M.F.)

2 Institut pour la Maîtrise de l'Énergie – Université d'Antananarivo BP 566, 101 Antananarivo, Madagascar; carine.chanthaw@gmail.com

3 Cardiff Catalysis Institute, School of Chemistry, Cardiff University, Main Building, Park Place, Cardiff, CF10 3AT, UK; SanchezF@cardiff.ac.uk (F.J.S.T.); MottaD@cardiff.ac.uk (D.M.)

4 Dipartimento di Chimica Industriale e dei Materiali, ALMA MATER STUDIORUM Università di Bologna, Viale Risorgimento 4, 40136 Bologna, Italy; tommaso.tabanelli@unibo.it

* Correspondence: nikolaos.dimitratos@unibo.it (N.D.); Alberto.villa@unimi.it (A.V.); Tel.: +39-051-209-3682 (N.D.); Tel.: +39-025-031-4361 (A.V.)

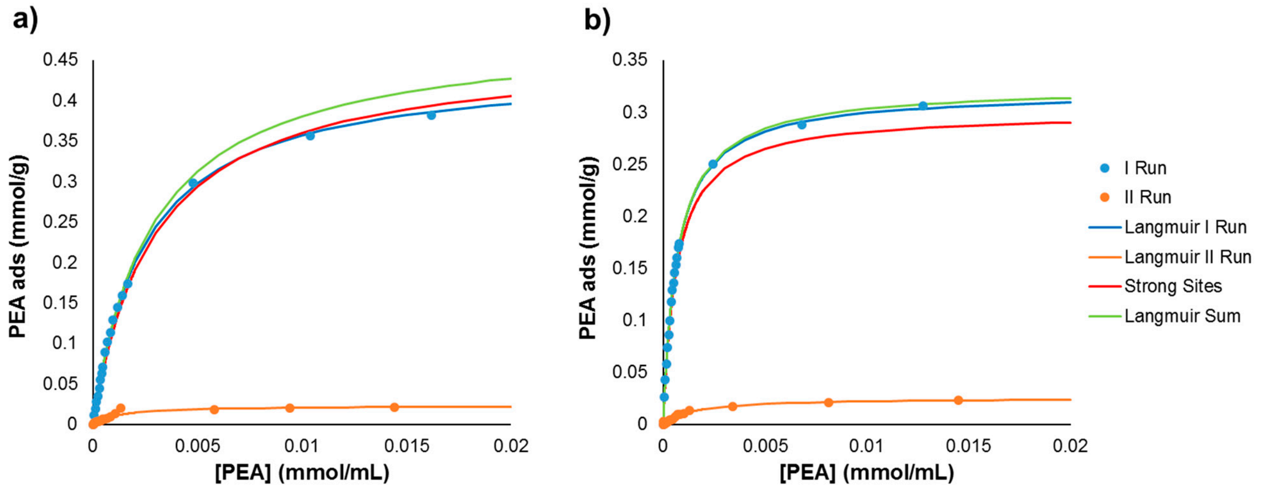

Figure S1. PEA adsorption isotherms, collected in cyclohexane at 30°C, for VPO (a) and Au-VPO (b). Experimental data were fitted using Langmuir model equation. Strong acid sites Langmuir isotherm was obtained by mathematical difference between first and second run of adsorption.

Table S1 Optimization of reaction parameters using 1%AuIW/VPO: Alcohol/Au ratio

Alcohol/Au ratio	Activity[1]	Selectivity (%)			
		Benzal-dehyde	Benzyl ether	Benzyl Benzoate	Benzoic acid
500	125	13	6	79	-
1000	120	8	6	76	-
2000	118	15	8	75	-

Reaction conditions. T = 120 °C, pO_2 = 2 atm, benzyl alcohol/xylene 25/75 per cent volume.

[1] Mol of alcohol converted per hour per total number of moles of Au calculated after 0.5 hour of reaction.

Table S2 Optimization of reaction parameters using 1%AuIW/VPO: pO_2

**pO_2 (atm)**	**Activity[1]**	**Selectivity (%)**			
		Benzal-dehyde	Benzyl ether	Benzyl Benzoate	Benzoic acid
1	114	12	8	74	-
2	120	8	6	76	-
3	116	9	7	76	-

Reaction conditions. Alcohol/Au ratio:1000 T = 120 °C, benzyl alcohol/xylene 25/75 per cent volume.

[1] Mol of alcohol converted per hour per mol of Au calculated after 0.5 hour of reaction.
